# Epidemiological Review on Monkeypox

**DOI:** 10.7759/cureus.34653

**Published:** 2023-02-05

**Authors:** Vashistha M Patel, Shreya V Patel

**Affiliations:** 1 Internal Medicine, Brookwood Baptist Health, Birmingham, USA

**Keywords:** zoonotic infections, west african clade, orthopoxvirus family, monkeypox virus, global epidemiology, central african clade

## Abstract

The significant increase in monkeypox cases that was reported at the beginning of 2022 was notable. The resurgence of viral zoonosis is especially concerning, given the current and recent COVID-19 epidemic. There are worries that a new pandemic may be beginning due to the virus that causes monkeypox spreading so quickly. This article aimed to provide an overview of the epidemiology, pathogenesis, and clinical symptoms of monkeypox. It has been known that monkeypox was primarily prevalent in Central and West Africa, but in recent years, cases of monkeypox infections have been reported around the world. The transmission of the infection to humans has been connected to exposure to a diseased animal or person's excretions and secretions. Various studies indicate that monkeypox clinically manifests as fever, fatigue, and a rash of smallpox-like lesions and can cause various complications, including pneumonia, encephalitis, and sepsis, which, when not properly managed, can lead to death. Those living in remote and forested areas, taking care of individuals infected with monkeypox, and trading and taking care of exotic animals are some of the risk factors for monkeypox. Men having sex with men are also at higher risk of contracting monkeypox. When dealing with individuals who have high-risk factors and come with new-onset progressive rashes, it is necessary for clinicians to highly suspect monkeypox. This review will serve as reference material and a supplement to the existing literature that will assist in the proper management and prevention of monkeypox.

## Introduction and background

Monkeypox (also known as Mpox) is a zoonotic orthopoxvirus that incidentally causes a smallpox-like illness in people, however, with noticeably reduced mortality. This virus is clinically relevant because it is native to Western and Central Africa, and incidences in the Western world have been connected to the exotic pet trade and foreign travel. Before smallpox's eradication and the subsequent lack of vaccination campaigns, vaccination offered coincidental immunity to the Mpox virus. Nevertheless, monkeypox's rise to clinical significance was made possible by these circumstances. This article describes the prevalence and pathogenesis of monkeypox and reviews the presentation of an individual infected with the Mpox virus with the motive to assist in the diagnosis, management, and prevention of Mpox disease.

The monkeypox virus, a member of the genus Orthopoxvirus, is responsible for the uncommon viral disease known as monkeypox. The Orthopoxvirus genus also comprises the smallpox-causing variola virus and the cowpox virus. The orthopoxvirus that causes monkeypox belongs to the poxviridae family. Poxviruses are brick-shaped and possess a lipoprotein-sheathed linear double-stranded deoxyribonucleic acid (DNA) genome [1,2]. Other than having the necessary replication, transcription, assembly, and egress proteins in their genome, poxviruses rely on host ribosomes for messenger ribonucleic acid (mRNA) translation [1,3]. Mpox and smallpox have some similarities. The smallpox virus, which is also categorized under the Orthopoxvirus family, has clinical signs and incubation time as Mpox [2]. However, the severity of Mpox is generally less, and the mortality rate lower than smallpox. The fact that many of the cases in the 2022 outbreak do not exhibit the typical signs of the monkeypox virus is one of its peculiarities. This makes it challenging to identify patients quickly and isolate them.

The term "monkeypox" was changed to "Mpox" in November 2022 by the World Health Organization (WHO) [4]. The original name was changed to avoid any potential stigma and to conform to modern best practices, which forbid disease names that are based on living things or places. Monkeypox virus will continue to be used to refer to the virus that causes Mpox until the International Committee on the Taxonomy of Viruses (ICTV) establishes what the virus's official name should be. However, the old Congo Basin (Central African) clade and the former West African clade were redesignated as Clade one (I) and Clade two (II), respectively. The WHO (2022) argues that the Congo Basin group is deadlier than the Western African clade. Subclades IIa and IIb make up Clade II.

## Review

Prevalence and incidence

Mpox was a zoonotic illness that was most prevalent in the Democratic Republic of the Congo and was indigenous to Central and Western Africa. A study suggested African rodents are the natural reservoir, even though the disease was initially discovered in captive monkeys (thus the name). Humans, mice, squirrels, monkeys, rats, and prairie dogs have all contracted the infection [5]. Two clades of the monkeypox virus that are genetically distinct from each another have been discovered. Compared to the West African clade, the Congo Basin (Central African) clade is reported more frequently and has documented incidences of human-to-human transmission [5].

Human Mpox incidences and occasional clusters have been identified outside of Africa. Centers for Disease Control and Prevention (CDC) documented that in 2003, Gambian giant rats were smuggled from Ghana and infected nearby prairie dogs that were on sale as pets in the Midwest of the United States. A total of 53 cases of Mpox in humans were the outcome of this [6]. An incident where three members of a family had visited Nigeria, returned to the UK in May 2021, and were later diagnosed with Mpox. The order of onset of symptoms in each case within the family (days 0, 19, and 33) may indicate a person-to-person transmission [7]. One case of the Mpox virus was found in the UK on May 7, 2022, and by June 8, 2022, 1,285 cases had been found in 28 different nations, as reported by WHO in 2022 [8]. CDC (2023) documents that 2,891 instances have been found in the US as of July 23, 2022 [9]. Precise prevalence and incidence figures are difficult to establish due to purported shortcomings in Mpox disease reporting and diagnosis. However, since routine smallpox immunization was stopped, both measures have grown [5].

Several risk factors correlate with Mpox infection. Another study discovered that individuals who live in densely forested and rural parts of Central and Western Africa, handle and prepare bush meat, provide care to others who are sick with the Mpox virus, and who have not had the smallpox vaccine are at risk for Mpox infection. The likelihood of infection has also been linked to the male gender. Society expects men to routinely hunt and interact with wild animals, and this predisposes them to infections [10]. A study concluded that there is currently a widespread Mpox outbreak affecting several nations on various continents, mostly in the community of men who have sex with men (MSM), with a presentation that primarily consists of genital lesions [11]. In another study, 99% of cases were found to be in the MSM community in a cohort of 595 Mpox-positive cases in Spain, with the lesions primarily affecting the vaginal, perineal, or perianal regions. Inguinal lymphadenopathy was also shown to be a common feature in the study, supporting the ideology that the main means of transmission were sexual intercourse [12]. As of July 2022, Germany reported 1,304 confirmed cases, mainly among MSM. Based on sequencing data from multiple nations, the West African clade of the Mpox virus is thought to be the cause of the 2022 outbreak [12-14]. There are worries that a new pandemic may be starting due to the virus that causes monkeypox spreading so quickly [6]. According to WHO (2022), one of the peculiarities of the current Mpox pandemic is that some cases have no history of visiting endemic regions or having contact with individuals from those regions. This suggests a potential unidentified chain of transmission that could aid in the spread of the Mpox virus [15]. The research found the mortality rate for the Central African variant to be 10.6%, compared to 3.6% for the Western African clade. From the study, the death rate for all cases was 8.7% [16]. A systematic analysis indicated that the mortality rate for those who had monkeypox ranged from 1% to 11% [17].

Transmission

Unlike the smallpox virus, the monkeypox virus also infects animals. Small mammals or monkeys seem to make up the majority of the afflicted animals [18]. It has been determined that a number of animals, including monkeys, rats, prairie dogs, and squirrels, have the illness or carry antibodies to the monkey virus. There is no conclusive information on the monkeypox virus's natural reservoir. The lack of virus isolates from animal species, including the serologically positive ones, makes the endeavor challenging.

Monkeypox is primarily transmitted to humans from infected animals, especially rodents and primates. The transmission of disease to humans has been linked to exposure to diseased animal secretions and excretions [5,19]. The monkeypox virus can transmit by a bite or scratch and cause a more serious sickness with a shorter incubation time. The spread of the monkeypox virus could be due to the international trade in exotic pets. Contact with sick prairie dogs was linked to an outbreak in the US in 2003. Through contact with imported rodents from Africa, the prairie dogs themselves contracted the disease** **[20-22]. This shows that the trade in exotic animals, particularly rodents, may be a significant factor in the spread of the monkey virus. In attempts to identify an animal reservoir, serology is troublesome for detecting the orthopoxvirus. Immunoglobulin M and immunoglobulin G are generated with other Orthopoxvirus species, making serology testing for these antibodies non-specific for the monkeypox virus [1]. Serology testing could very well be picking up orthopoxvirus in addition to the monkeypox virus. Since so few viruses have been isolated from the various animal species, the search for the monkey virus's natural reservoir has become more challenging.

The virus can also be transmitted from one person to another through close contact with infected respiratory secretions, such as saliva or mucus, or through contact with skin lesions or other bodily fluids. Sharing drinking cups, sharing food, and sharing close quarters for sleeping were all linked to higher transmission risks. The virus appears to propagate largely through oral mucosae [23]. WHO (2022) mentions that Mpox proliferation is unique, given that gay people account for the majority of the sources for the 2022 monkeypox virus. This significance of the gastrointestinal tract and mouth mucosa may help to explain why gay people were disproportionately affected by the 2022 outbreak of the monkey virus. Money pox typically takes six to 13 days to incubate, but it can take anything from five to 21 days. The method of transmission may significantly impact the virus's clinical symptoms and spread [15].

Pathogenesis

After viral entrance by any route (oropharynx, nasopharynx, or intradermal) into the body, it infects and replicates in cells of the immune system, particularly dendritic cells and macrophages [24]. These cells then migrate to lymph nodes and begin to infect local endothelial cells, leading to the formation of small blood clots. The virus then spreads to other tissues and organs through the bloodstream, causing a wide range of symptoms. This is considered the incubation period, which can last up to 21 days and usually lasts seven to 14 days. This means the symptoms of monkeypox typically appear within seven to 14 days after infection. Prodromal symptoms appear from one to two days before lesions could be seen, and these include headache, myalgia, backache, chills, lethargy, lymphadenopathy, and fever caused by secondary viremia. After that, a rash appears, frequently first on the face before moving to other regions of the body. This rash evolves and passes through various stages before becoming a scab and then healing. Patients who are sick now might spread the virus. The development of skin lesions begins at the oropharynx. As the virus continues to replicate, it causes inflammation and damage to the skin, respiratory system, and other organs. In severe cases, monkeypox can lead to pneumonia, encephalitis, or septicemia, which can be fatal [25]. Serum antibodies are frequently detectable by the time lesions begin to develop [26].

Overall, the immune system's response to the virus also plays a huge role in the course of monkeypox infection; severe or mild symptoms could be a result of the immune system's response [25]. Also, recovery from monkeypox provides immunity to the virus, but it is not known to be long-lasting. The B10R, B14R, B19R, D10L, and D14L genes may be in charge of the monkeypox virus's pathogenicity [27]. An investigation of the Central African clade revealed that, with the remarkable exception of the interleukin 1 beta gene, the majority of the changes occurred in the non-coding area of the genome. The mutation causes a reduction in cytokine binding and a weakening of the immune response. The development of the monkeypox virus and the severity of the illness may be significantly influenced by the transmission mechanism. The severity of the disease may also be influenced by mutations in the CAR15c/18c sequences [28]. The monkeypox virus-infected cells were poorly identified by host antiviral CD4+ and CD8+ lymphocytes. Monocyte infection by the monkeypox virus has also been demonstrated. An immunoregulator protein that inhibits immune response appears to be produced in greater quantities by the monkeypox virus. Recombination often occurs in poxviruses and can lead to quickly mutating viruses [1]. This raises the likelihood that new strains will emerge, some of which may be more deadly or contagious than the original. Western monkeypox clade is thought to be less deadly than the Central clade. Concurrent infection and lowered immunity may worsen monkeypox [29].

The pathogenesis of the virus has also changed as a result of genetic mutations and alterations. According to meta-analysis research, children were primarily impacted by the monkeypox virus in the 1970s. The average age of those affected has risen as a result of genetic mutation, and in the year 2010, 21 was reported to be the average age of those infected. The monkeypox virus is undergoing fast alterations that could make it more dangerous and lethal [16].

Clinical manifestations

The initial symptoms of Mpox include fever, body aches, lymphadenopathy, and headache. The face and surrounding areas are more severely impacted than the trunk. Mouth and vaginal mucosal membranes could potentially be affected. Mouth mucosal lesions appear after one to two days, immediately followed by skin lesions on the face and extremities, including palms and soles. These lesions are centrifugally concentrated. There may or may not be any spread of the rash to the rest of the body. The lesions may range from a few to thousands in number. The lesions progress through macular, papular, vesicular, and pustular phases during the next two to four weeks. Lesions are described as hard, deeply seated, and 2 to 10 mm in size, changing synchronously. Before the formation of crusts, lesions stay in the pustular phase for five to seven days. Over the following seven to 14 days, crusts develop and desquamate, and the illness clears itself three to four weeks after the beginning of symptoms in many cases. Once all of the crusts have fallen off, the patient is no longer regarded as contagious [24].

In comparison to other illnesses like chicken pox, the monkeypox lesions take longer to turn into crusts. Additionally, the lesions might range from one to thousands. In a relatively small number of individuals, the corneal and conjunctival mucosa were affected. Lymphadenopathy is regarded as a defining characteristic of the monkeypox virus and can be used to distinguish monkeypox from other pox illnesses [24,30]. The CDC provides weekly statistics on the prevalence of the symptoms of Mpox as reported. In one of its reports, CDC mentioned that the signs and symptoms that were most frequently recorded were rash (100%), fever (63%), chills (59%), and lymphadenopathy (59%). Purulent or bloody feces (21%), rectal pain (22%), and rectal bleeding were among the rectal symptoms that were reported [31].

Up to 58% of the 291 people who were aware of their first symptoms available reported having at least one prodromal symptom; the illness started with a rash for 42% of individuals. The most common body parts with rash were the genitalia (46%), arms (40%), face (38%), and legs (37%); of the 718 Mpox patients who reported rash, 238 (33%), 126 (18%), 98 (14%), and 256 (36%) had rash in one, two, three, or more body locations. Another study found that mouth sores, vomiting, and hospital stay longer than 48 hours were all independently correlated with these symptoms [32]. The MSM community is especially vulnerable to the current outbreak. Umbilicated, pseudo-pustular, and vesicular lesions on the skin, together with fever, weakness, exhaustion, headaches, and regional lymphadenopathy, are the primary clinical features. Lesion clustering and frequent involvement of the genital or perianal region are regarded to be correlated to the sexual nature of transmission [33-34].

Monkeypox has been associated with various complications. In severe cases, monkeypox can lead to complications such as pneumonia, encephalitis, and sepsis. Secondary bacterial infections, such as skin infections, can arise in monkeypox patients and cause serious disease or even death. This is especially true for persons with compromised immune systems, including those with HIV/AIDS. Rarely, individuals with monkeypox may experience a severe immunological response to the virus, described as a "cytokine storm," in which the body is severely inflammatory and damaged [25,35]. It's crucial to note that, with the right care and treatment, the majority of monkeypox victims recover, with only 1-10% passing away as a result of the infection [36]. Monkeypox, however, can sometimes be fatal in children and those with compromised immune systems, such as those with HIV/AIDs.

## Conclusions

Monkeypox is a rare viral disease that occurs majorly in Central and West Africa. Over recent years, cases of Mpox infections have been reported around the world. It is transmitted to humans from infected animals and can also be transmitted from person to person. The transmission of disease to humans has been linked to exposure to a diseased animal or person's excretions and secretions. The disease is characterized by fever, fatigue, and a rash of smallpox-like lesions, and it can lead to complications such as pneumonia, encephalitis, and sepsis which may be fatal. Monkeypox's quick spread has exposed the inadequacy of precautions used to stop outbreaks. Animals being transported internationally must be quarantined and tested for the monkeypox virus. When examining patients, particularly the high-risk population, a strong index of suspicion must be in place. Mpox vaccination should be started in high-risk groups such as the immunocompromised, MSM, and people who handle animals that have traveled internationally, particularly rodents and other small mammals. To make an early diagnosis and start quick treatment, doctors need to be conscious of the early clinical manifestations of the monkeypox virus. Until all lesions have healed, patients with proven or suspected monkeypox infections must be quarantined. More study is required to develop more efficient preventive and treatment methods.
